# Popular Treatment of Cholera

**Published:** 1866-02

**Authors:** M. L. North

**Affiliations:** Saratoga Springs


### POPULAR TREATMENT OF CHOLERA.

"Suppose our profession should arouse and make a combined movement to help the community to an accurate discrimination of the disease in its early stage. Why don't our editors instruct the public? The distinction between Asiatic Cholera and common domestic diarrhœa is palpable and easy, and every man can carry that distinction in his memory. Cannot an uneducated man tell certainly if he has an evacuation which is *copious*, *watery*, *colorless*, *painless*, and *inodorous?* Any man of ordinary talents can ascertain, in two minutes, that something has happened to him which he never experienced before. I said *painless.* It is this quality of the evacuation which leads men to the amazing apathy so common, and permits them to let hours, even days elapse before the physician is at his post.

As this Asiatic destroyer may become Americanized, our people *must* be able to make an early discrimination, and our profession *must* learn how to prevent the fatal collapse. Why will not the editors instruct their readers that they can better afford to lose a pint of common red blood than a pint of this colorless blood of cholera? How hopeless is the state of the patient from whom gallons of liquid, colorless nutriment have escaped!

If the editors, and especially my medical brethren, could feel as I do on the subject of incipient cholera, and lend us their facts and thoughts through the medical journals, in short, condensed paragraphs, my hopes would be answered.

Having been watching every movement since this disease first broke out near Calcutta, in 1817, I have seen no scheme so rational as that fixed on by the Army Board of Surgeons of Bengal, and, according to reports, more successful when taken in the early stage. It consisted of heroic doses of calomel, combined with opium sufficient to anchor the calomel and retain it in the bowels. The formula was a combination of 15 grains of calomel and 4 grains of opium. Possibly it was five grains of opium. Fifteen or twenty grains of calomel every four hours, with opium only sufficient to control the bowels, must have a powerful and rapid effect in changing the secretions. But if every business man would keep a powder of the above description in his pocket to swallow if occasion required, it would scarcely do harm, and would greatly aid the efforts of the physician employed."

M. L. NORTH.

*Saratoga Springs.*

